# A Comparative Study of the Clinical Benefits of Rivaroxaban and Warfarin in Patients With Non-valvular Atrial Fibrillation With High Bleeding Risk

**DOI:** 10.3389/fcvm.2022.803233

**Published:** 2022-02-16

**Authors:** Peng-Hui Liu, Ze-Hua Liu, Ming-Hui Niu, Ping Chen, Yuan-Bin Shi, Fei He, Rong Guo

**Affiliations:** ^1^Department of Cardiology, The First Affiliated Hospital of Zhengzhou University, Zhengzhou, China; ^2^Department of Hematology, The First Affiliated Hospital of Zhengzhou University, Zhengzhou, China

**Keywords:** warfarin, non-valvular atrial fibrillation, clinical benefits, high bleeding risk, rivaroxaban

## Abstract

**Objective:**

To compare the clinical benefits of rivaroxaban and warfarin in patients with non-valvular atrial fibrillation (NVAF) with high bleeding risk.

**Methods:**

A retrospective study was conducted on patients with high bleeding risk NVAF who were hospitalized at the First Affiliated Hospital of Zhengzhou University between May 31, 2016 and May 31, 2019 and took at least rivaroxaban and warfarin. The clinical benefits of both drugs were assessed by efficacy benefit and safety risk. The primary efficacy benefit was a composite end point for stroke (both ischemic and hemorrhagic) and systemic embolism. The secondary efficacy end points were death and myocardial infarction (MI). The principal safety end point was the composite end point of fatal bleeding and critical organ bleeding.

**Results:**

A total of 1,246 patients with high bleeding risk were enrolled, including 787 patients in the rivaroxaban group and 459 patients in the warfarin group. Results of the primary efficacy benefit endpoint were obtained from 104 patients (13.2%) in the rivaroxaban group and 88 (19.2%) patients in the warfarin group (hazard ratio [HR]: 0.681; 95% confidence interval [CI]: 0.512–0.906; *P* < 0.001 for non-inferiority). The principal safety end points were observed in 49 (6.23%) patients in the rivaroxaban group and in 55 (11.98%) patients in the warfarin group (HR: 0.469 in the rivaroxaban group; 95% CI: 0.314–0.702; *P* < 0.001). With respect to secondary efficacy and benefit endpoints, 28 (3.56%) patients in the rivaroxaban group and 22 (4.79%) patients in the warfarin group died, with an HR of 0.760 (95% CI: 0.435–1.329; *P* = 0.336); 32 (4.07%) patients in the rivaroxaban group; and 26 (5.66%) patients in the warfarin group had MI, with an HR of 1.940 (95% CI: 0.495–1.069, *P* = 0.254) in the rivaroxaban group.

**Conclusions:**

Rivaroxaban is non-inferior to warfarin in the prevention of stroke and systemic embolism in patients with high blood NVAF. Rivaroxaban is superior to warfarin in reducing fatal bleeding and bleeding in critical organs.

**Clinical Trial Registration:**

Chinese Clinical Trials Registry, identifier ChiCTR2100052454.

## Introduction

Patients with atrial fibrillation (AF) have a higher incidence and risk of embolism, with a 4–5-fold increased risk of ischemic stroke. The proportion of stroke caused by AF increases with age; overall, the proportion is about 10–24% in persons aged between 80 and 89 years (1–3). Current professional society guidelines recommend treatment with an oral anticoagulant for patients with AF who are at an increased risk of thromboembolism (4–6). Past use of warfarin has significantly reduced the risk of stroke in patients with AF. Furthermore, warfarin treatment has a narrow therapeutic range, interacts with food and other drugs, and requires regular international normalized ratio (INR) monitoring and frequent dose adjustments (7–9). In recent years, rivaroxaban has been approved for stroke prevention in AF in randomized controlled trials, owing to its non-inferiority in both efficacy and safety when compared to warfarin. In addition, anticoagulant monitoring is not required, and the significant reduction in drug-food interactions also changes the prospects for stroke prevention in AF (10).

Several studies have comparatively analyzed the clinical benefits of rivaroxaban and warfarin (11–16). Manesh et al. (11) conducted a prospective randomized controlled trial—ROCKET-AF—including 14,264 patients with non-valvular atrial fibrillation (NVAF) from 1,178 medical centers across 45 countries. The results showed that rivaroxaban was non-inferior to warfarin in the prevention of stroke or non-central embolism. Furthermore, the incidences of intracranial hemorrhage and fatal hemorrhage were lower in the rivaroxaban group than the warfarin group; no significant difference in the risk of massive hemorrhage was observed between the two groups. However, their study included only a relatively small number of Asian patients and did not differentiate between high and low bleeding risk. Hence, Lee et al. (12) compared the safety and effectiveness of oral anticoagulants in Asian patients with NVAF, and the results showed that rivaroxaban was associated with a lower incidence of ischemic stroke and major bleeding than warfarin. However, patients with prior stroke, cerebral hemorrhage, or gastrointestinal bleeding were not included in the study, and the results of the study could not be extrapolated to patients with high bleeding risk. The ARISTOPHANES study, the largest retrospective observational study conducted by Gyh et al. (13), showed that rivaroxaban had a lower incidence of stroke and systemic embolism than warfarin. Although the study further analyzed various subgroup indicators, such as heart failure, cerebral infarction, CHADS2-VASc score, and HAS-BLED score, the results were not explained in detail. Further, the study population was entirely from the United States; hence, data on Asian patients with high bleeding risk were not available, which was likely relevant to the final results (17, 18). The Centers for Disease Control and Prevention reported a higher intracranial hemorrhage (ICH) mortality among Asians compared with whites (19). The reason is multifactorial; genetically, Asian patients are more likely to be warfarin sensitive or highly sensitive responders, who seem prone to excessive bleeding (20). Except for the variations in distributions of genetic polymorphisms for warfarin metabolism (21, 22), Asian patients tended to have lower body weight, smaller proportions of prior myocardial infarction (23, 24) etc. However, in clinical practice, patients on Asian with AF with multiple complications tend to have high bleeding risk. In 2010, The European Society of Cardiology (ESC) guidelines for the treatment of AF introduced for the first time the HAS-BLED bleeding risk assessment program (25). Therefore, this study specifically conducted a comparative analysis on Asian patients with AF with high bleeding risk.

## Methods

The study data were collected from 146,413 patients hospitalized between May 31, 2016 and May 31, 2019 in 11 wards (including 2 critical care units) of the Department of Cardiology at The First Affiliated Hospital of Zhengzhou University, Heyi, and Zhengdong Hospital. Among these, patients with AF were screened out. The inclusion criteria were as follows: (1) Dynamic electrocardiogram was in line with the diagnostic criteria of AF; (2) imaging diagnosis excluded valvular disease; (3) meeting the diagnostic criteria for high risk of bleeding (HAS-BLED score ≥3 points); (4) Those requiring routine anticoagulant therapy with warfarin or rivaroxaban (risk stratification score ≥1 according to CHADS2-VASC); and (5) age ≥18 years. The following patients were excluded: (1) Those that did not comply to treatment regimen; (2) those lost to telephonic follow-up; and (3) those who switched to other types of oral anticoagulant medication during follow-up.

Study participants were divided into two groups: (i) Rivaroxaban group, rivaroxaban (Bayer Healthcare Co., LTD., National drug approval J20180077, 20 mg × 7 tablets) was used for anticoagulant therapy. Fixed oral doses of 15 mg or 20 mg *per session* were given at fixed times of the day, depending on the patient's creatinine clearance. (ii) Warfarin group: warfarin (Qilu Pharmaceutical Co., LTD., National drug approval H37021334, 2.5 mg × 60 tablets) was used for anticoagulant therapy, and the initial dose was controlled at 2.5 mg/d. Coagulation indices were monitored before medication, and the INR values were measured. The INR values were tested regularly while the patients were on medication, and the values were controlled between 2.0 and 3.0.

A total of 1,343 patients with NVAF who took at least one anticoagulant drug were enrolled between the above-mentioned dates. Each patient was followed-up for 730 days from the beginning of medication by inquiring about hospitalization case, medical advice, and telephone contact. Endpoints related to all-cause death, stroke, MI, systemic embolism, and fatal and critical organ bleeding were recorded during drug administration. Eighteen cases were lost to follow-up, 48 cases did not adhere to the prescribed medication, and 31 cases changed to other types of anticoagulants that were excluded from the study scope. The number of patients eligible for the study was 1,246.

### Study Endpoints

The primary efficacy benefit end point of this retrospective study was defined as the composite end point of stroke (ischemic and hemorrhagic) and systemic embolism, and the secondary efficacy benefit end point was defined as all-cause death and MI. The primary safety risk end point was defined as major bleeding, including composite end point of fatal or critical organ bleeding. Fatal bleeding was defined as whole blood transfusion or erythrocyte concentration ≥2 units or hemoglobin reduction ≥2 g/dL. Bleeding in critical organs was defined as bleeding from any of the following anatomical sites: intracranial, spinal, ocular, pericardial, joint, retroperitoneal, or muscular compartment syndrome. Bleeding events involving the central nervous system that met the definition of stroke were considered as hemorrhagic strokes and included in both the primary efficacy and safety end points.

### Statistical Analysis

Normality test was performed on the measurement data. Normally distributed data were represented as mean ± SD; non-normally distributed data were represented as medians with interquartile ranges (IQR). Classified data were expressed as percentages. Independent-sample *t*-test or Mann–Whitney *U* test was used for pairwise comparison, and the chi-square test was used for comparison of rates. The primary efficacy end point was determined to be a non-inferiority margin of 1.46 by the non-inferiority test. The risk ratios, confident intervals (CIs), and *P*-values of efficacy end points and safety end points were calculated using the multivariate Cox proportional risk model, and treatment drugs were considered the only co-variable. *P* ≤ 0.05 indicated statistical difference. By plotting the cumulative rate of events over time, the difference between the occurrence of two sets of data events was apparent.

## Results

The main clinical characteristics of the patients included in the analysis are shown in Table 1. Corresponding statistical methods were used to compare the baseline data of the two groups. Most clinical characteristics were similar between the two groups.

**Table 1:** Baseline characteristics of the study population (*N* = 1246).

**Characteristic**	**Rivaroxaban (*n* = 787)**	**Warfarin (*n* = 459)**	***T*-test or chi-square test**	***P*-value**
Age (years), mean ± SD	71.23 ± 7.73	70.51 ± 7.70	1.604	0.109
Male sex, *n* (%)	461 (58.6)	268 (58.4)	0.004	0.948
**^1^CHADS2-VASc score (*****n*** **%)**				
1	105 (13.34)	65 (14.16)	0.172	0.678
2	170 (21.60)	97 (21.13)	0.038	0.846
3	203 (25.79)	113 (24.62)	0.212	0.646
4	188 (23.89)	102 (22.22)	0.451	0.502
5	73 (9.28)	48 (10.46)	0.462	0.497
6	38 (4.83)	28 (6.10)	0.935	0.334
7	10 (1.27)	6 (1.31)	0.003	0.956
**^2^HAS-BLED score (*****n*** **%)**				
3	690 (87.67)	397 (86.49)	0.364	0.546
4	86 (10.93)	55 (11.98)	0.322	0.571
5	10 (1.27)	7 (1.53)	0.139	0.709
6	1 (0.13)	0 (0.00)	0.584	0.445
Current baseline characteristics, Mean ± SD				
^3^BNP	2463.29 ± 3965.38	2636.53 ± 4266.04	0.723	0.470
^4^EF	56.68 ± 9.95	56.08 ± 10.42	1.014	0.311
Systolic blood pressure	162.33 ± 30.63	157.75 ± 30.91	2.541	0.081
Diastolic blood pressure	93.03 ± 18.69	91.47 ± 18.69	1.424	0.155
Alanine aminotransferase	29.60 ± 39.71	29.81 ± 45.83	0.079	0.937
Aspartate aminotransferase	29.19 ± 38.59	30.92 ± 49.02	0.686	0.493
Direct bilirubin	6.92 ± 5.42	6.95 ± 5.22	0.113	0.910
Indirect bilirubin	6.98 ± 5.86	7.99 ± 5.39	3.028	0.003^*^
Alkaline phosphatase	82.24 ± 62.22	77.76 ± 45.80	1.344	0.179
Creatinine	84.51 ± 42.85	96.85 ± 72.33	3.776	0.000^*^
Hemoglobin	132.59 ± 18.49	130.87 ± 19.30	1.558	0.119
INR	1.53 ± 6.58	1.42 ± 0.74	0.375	0.707
**Medical history (** ***n*** **%)**				
Heart failure	245 (31.1)	122 (26.6)	2.890	0.089
Diabetes	153 (19.4)	92 (20.0)	0.067	0.796
Hypertension	583 (74.1)	313 (68.2)	4.974	0.026^*^
Stroke	138 (17.5)	90 (19.6)	0.833	0.361
Thromboembolism^5^	21 (2.7)	13 (2.8)	0.029	0.864
^6^TIA	35 (4.4)	21 (4.6)	0.011	0.916
Vascular disease^7^	37 (4.7)	23 (5.0)	0.061	0.816
History of nonsteroidal drug use	397 (50.4)	211 (46.0)	2.324	0.127
Smoking	245 (31.1)	132 (28.8)	0.773	0.379
Alcohol	379 (48.2)	236 (51.4)	1.232	0.267

1 *CHADS2-VASc, congestive,heart,failure, hypertension, age75(doubled), diabetes mellitus, stroke(doubled)-vascular disease, age65-74 and sex category(female)*.

2 *HAS-BLED, hypertension, abnormal renal and liver function, stroke, bleeding, labile INRs, elderly, drugs and alcohol*.

3 *BNP, brain natriuretic peptide*.

4 *EF, ejection fraction*.

5 *Thromboembolism includes pulmonary embolism, organ embolism, and lower limb embolism*.

6 *TIA, transient ischemic attack*.

7 *Vascular disease includes peripheral artery disease, MI, and complex aortic plaques*.

* *indicates the significant values*.

**Figure 1:**
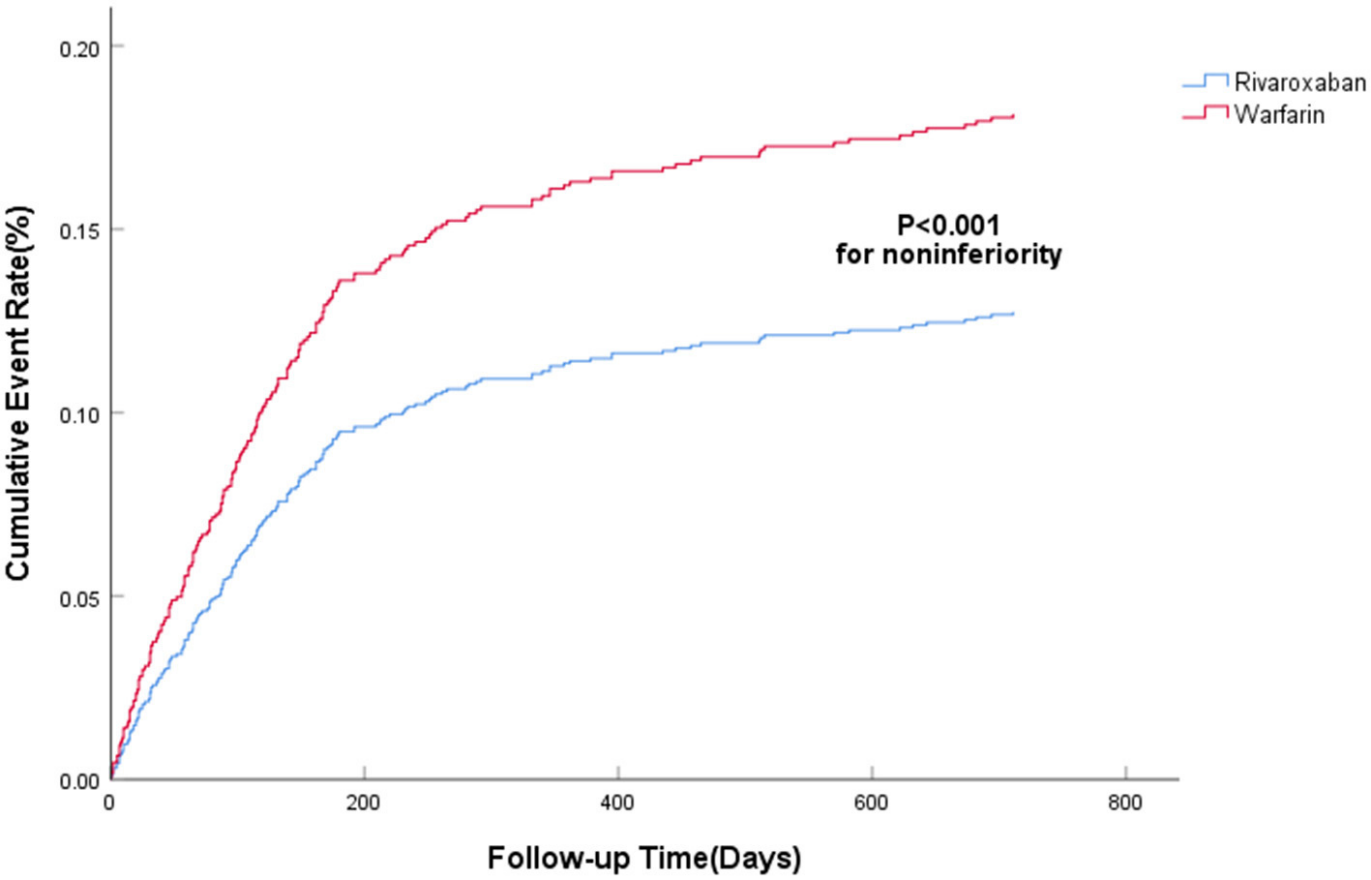
The primary efficacy benefit end point.

**Figure 2:**
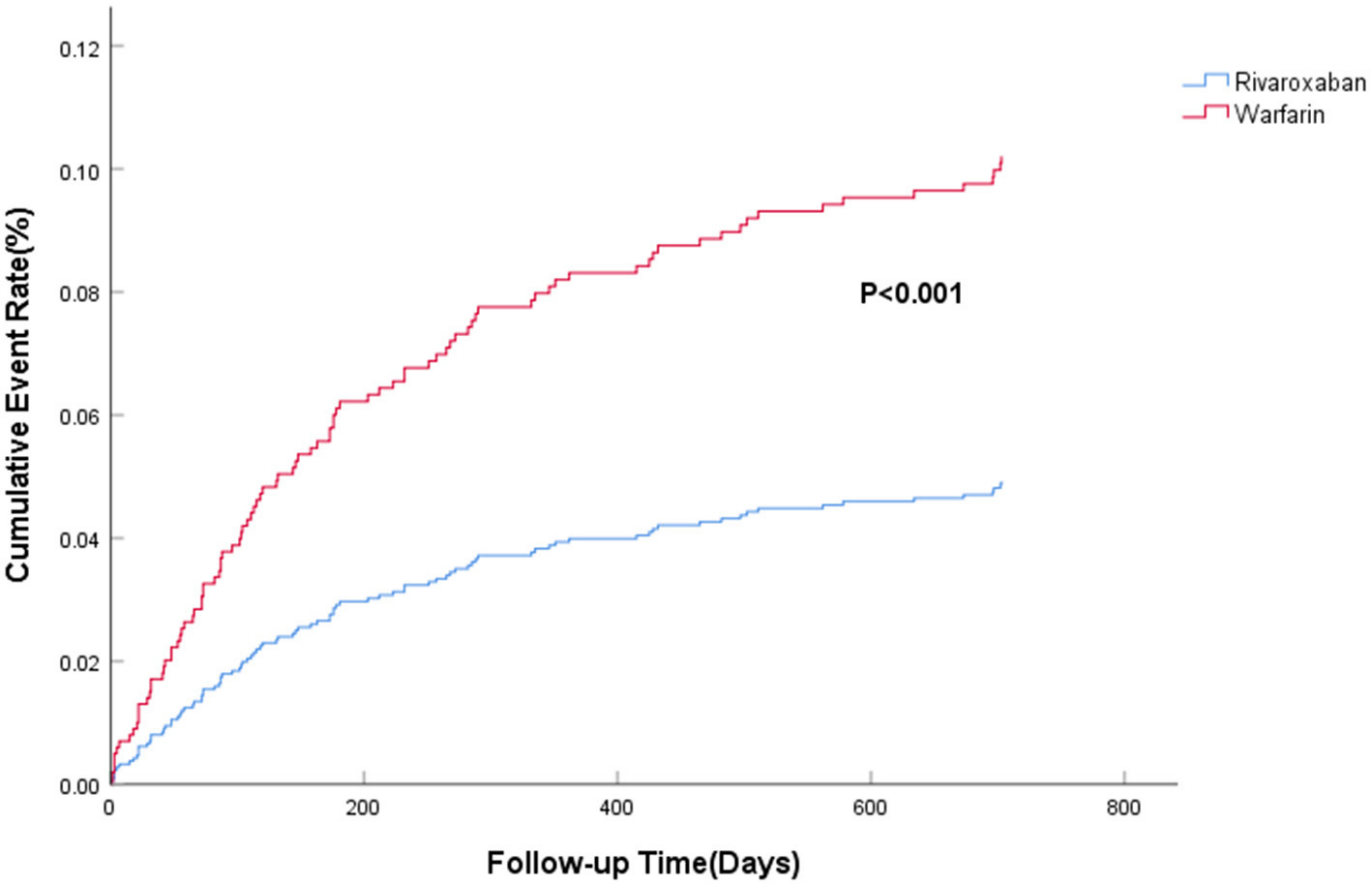
The primary safety risk end point.

The efficacy benefit and safety end points in this study are shown in Table 2. The principal efficacy benefit end points were obtained from 104 cases (13.2%) in the rivaroxaban group and 88 cases (19.2%) in the warfarin group, and the hazard ratio (HR) of the rivaroxaban group was 0.681 (95% CI: 0.512–0.906; *P* for non-inferiority <0.001), suggesting that rivaroxaban was non-inferior to warfarin in preventing stroke and non-central embolism in this population with high bleeding risk, as shown in Figure 1. The principal safety end points were observed in 49 (6.23%) patients in the rivaroxaban group and 55 (11.98%) patients in the warfarin group, with an HR of 0.469 (95%CI: 0.314–0.702; *P* < 0.001), as shown in Figure 2. Fatal bleeding occurred in 21 patients in the rivaroxaban group (2.67%) and 25 patients in the warfarin group (5.45%), with an HR of 0.530 (95% CI: 0.287–0.979; *P* = 0.043). Furthermore, 28 (3.56%) patients in the rivaroxaban group and 20 (6.54%) patients in the warfarin group had critical organ bleeding (HR: 0.484 in the rivaroxaban group, 95% CI: 0.290–0.808; *P* = 0.005). Analysis of ICH events showed that 18 (2.29%) ICH events were recorded in the rivaroxaban group and 20 (4.36%) ICH events in the warfarin group, and the HR of the rivaroxaban group was 0.249 (95% CI: 0.139–0.448; *P* < 0.001), indicating that rivaroxaban was more advantageous than warfarin in reducing fatal bleeding and critical organ bleeding. The difference in critical organ bleeding was mainly due to ICH.

**Table 2:** Comparison of end point events in the rivaroxaban and warfarin groups.

**Clinical outcome**	**All (*N* = 1246, n%)**	**Rivaroxaban (*n* = 787)**	**Warfarin (*n* = 459)**	**Multivariable adjustment OR (95%CI)**	**P or P for non-inferiority value**
The primary efficacy endpoint	192 (15.41)	104 (13.21)	88 (19.17)	0.681 (0.512–0.906)	<0.001^1*^
Stroke^2^	105 (8.43)	57 (7.24)	48 (10.5)	0.728 (0.495–1.069)	0.105
MI^3^	58 (4.65)	32 (4.07)	26 (5.66)	0.738 (0.438–1.243)	0.254
PTE^4^	19 (1.52)	10 (1.27)	9 (1.96)	0.639 (0.260–1.572)	0.330
Lower-extremity thrombosis	33 (2.65)	16 (2.03)	17 (3.70)	0.589 (0.295– 1.499)	0.135
All-cause death	50 (4.01)	28 (3.56)	22 (4.79)	0.760 (0.435–1.329)	0.336
Primary safety endpoint	104 (8.35)	49 (6.23)	55 (11.98)	0.469 (0.314–0.702)	<0.001*
Fatal^5^ bleeding	49 (3.93)	21 (2.67)	25 (5.45)	0.530 (0.287–0.979)	0.043*
Critical organ bleeding^6^	58 (4.65)	28 (3.56)	30 (6.54)	0.484 (0.290–0.808)	0.005*
ICH^7^	38 (3.05)	18 (2.29)	20 (4.36)	0.249 (0.139–0.448)	<0.001^*^

1 *P for non-inferiority < 0.001*.

2 *Both ischemic and hemorrhagic stroke*.

3 *Myocardial infarction*.

4 *Pulmonary thromboembolism*.

5 *Whole blood transfusion or erythrocyte concentration ≥2 units or hemoglobin reduction ≥2 g/dL*.

6 *Bleeding from any of the following anatomical sites: intracranial, spinal, eye, pericardium, joint, retroperitoneal, or muscular compartment syndrome*.

7 *Intracranial hemorrhage*.

* *indicates the significant values*.

Secondary efficacy and benefit endpoints: Overall, 28 (3.56%) patients in the rivaroxaban group and 22 (4.79%) in the warfarin group died, with an HR of 0.760 (95% CI: 0.435–1.329; *P* = 0.336); 32 (4.07%) patients in the rivaroxaban group and 26 (5.66%) patients in the warfarin group had MI, with an HR of 1.940 (95% CI: 0.495–1.069, *P* = 0.254) in the rivaroxaban group. This showed no significant difference between the rivaroxaban and warfarin groups in the prevention of all-cause death and MI.

## Discussion

In this retrospective study, we evaluated the clinical benefits of rivaroxaban and warfarin by comparing efficacy benefits and safety risks. Rivaroxaban was non-inferior to warfarin in preventing stroke and non-central embolism. The most worrisome complication of anticoagulation is bleeding, and the low incidence of fatal or critical anatomic bleeding in the rivaroxaban group supports its clinical use. ROCKET-AF and other related large studies concluded that rivaroxaban is non-inferior to warfarin in terms of efficacy and benefit in patients with AF (11). Rivaroxaban has a similar overall risk of major bleeding as warfarin. However, in the subcategory of major bleeding, rivaroxaban was associated with a lower incidence of fatal bleeding and intracranial hemorrhage, but no significant difference in the risk of major bleeding was observed between the two groups (13, 26, 27). However, ROCKET-AF data were collected from 45 countries and did not differentiate between high and low risk of bleeding. The results obtained in our study show that these conclusions are applicable to Asian populations with higher risk of bleeding, which provides additional evidence for clinical use.

Analysis of these events individually showed no significant difference between the two anticoagulants in terms of death and MI. This observation may be due to the small sample size, which was not enough to show a difference. In addition, according to the ROCKET-AF test, most deaths (72%) were due to cardiovascular disease, whereas only 6% of deaths were due to non-hemorrhagic stroke or systemic embolism. Therefore, although more fatal or critical organ bleeding events were recorded in the rivaroxaban group, no significant increase in all-cause deaths was observed.

Rivaroxaban showed obvious advantages regarding fatal bleeding, likely because warfarin dose is easily affected by food and other drugs. Rivaroxaban, on the contrary, is taken orally in a fixed daily dose and is not affected by food or other drugs. Moreover, the drug levels of rivaroxaban do not fluctuate with the effect of external factors; hence, it shows a significant advantage over warfarin (28). In addition, possibly, some patients on warfarin therapy did not regularly test their INR values, as recommended after discharge (29), which led to poor control of INR values and increased fatal bleeding events. In terms of critical organ bleeding events, the incidence of events in the rivaroxaban group was significantly different from that in the warfarin group, which was mainly due to the significant reduction in ICH events. These findings were consistent with the results of previous randomized clinical trials.

Our research participants comprised all inpatients treated in our hospital, and all the data were obtained from the hospital medical records system and patient follow-up, which is very similar to the actual situation of clinical treatment. This study provides supplementary evidence for clinical trial results, and provides reference for the clinical practice of drug use.

This retrospective observational study had several limitations. First, our study was constrained by the inherent limitations of a retrospective evaluation of previous data. Hence, only statistical associations could be drawn; causation could not be determined. Second, although possible influencing factors and several comorbidities were adjusted for by multivariate COX regression, potential residual confounders remained. In clinical practice, systemic differences in patients receiving different oral anticoagulants may be present, and if such differences are not observed, the study results may be biased. Therefore, the possibility that the observed correlations could be attributed to factors not considered in our model cannot be ruled out. Third, our data sources were from inpatients in the Department of Cardiology of the First Affiliated Hospital of Zhengzhou University, and the conclusions were most common only to patients in Asians and further research is needed to confirm whether this conclusion is applicable to non-Asian populations.

Finally, given the large number of statistical tests, especially the cross-tests, the possibility of type I errors cannot be ruled out.

## Conclusion

Overall, rivaroxaban was non-inferior to warfarin in preventing stroke and systemic embolism in patients with NVAF with high bleeding risk in this study. Rivaroxaban is superior to warfarin in reducing fatal bleeding and bleeding in critical organs.

## Data Availability Statement

The raw data supporting the conclusions of this article will be made available by the authors, without undue reservation.

## Ethics Statement

Ethical review and approval was not required for this study with human participants, in accordance with the local legislation and institutional requirements. Written informed consent was not required for this study, in accordance with the local legislation and institutional requirements.

## Author Contributions

FH and P-HL conceived, designed, and planned the study. P-HL analyzed the data. P-HL and RG interpreted results. P-HL, FH, and RG drafted the report. RG, Z-HL, M-HN, PC, and Y-BS were involved in the critical revision of the manuscript. All authors contributed to the article, data acquisition, and approved the submitted version.

## Funding

This work was supported by Science and Technology Plan of Henan Province (No. 182102310160), Medical Science and Technology Research Project of Henan Province (2018020118).

## Conflict of Interest

The authors declare that the research was conducted in the absence of any commercial or financial relationships that could be construed as a potential conflict of interest.

## Publisher's Note

All claims expressed in this article are solely those of the authors and do not necessarily represent those of their affiliated organizations, or those of the publisher, the editors and the reviewers. Any product that may be evaluated in this article, or claim that may be made by its manufacturer, is not guaranteed or endorsed by the publisher.
